# Condensed Tannins from *Ficus virens* as Tyrosinase Inhibitors: Structure, Inhibitory Activity and Molecular Mechanism

**DOI:** 10.1371/journal.pone.0091809

**Published:** 2014-03-17

**Authors:** Xiao-Xin Chen, Yan Shi, Wei-Ming Chai, Hui-Ling Feng, Jiang-Xing Zhuang, Qing-Xi Chen

**Affiliations:** 1 Key Lab of the Ministry of Education for Coastal and Wetland Ecosystems, School of Life Sciences, Xiamen University, Xiamen, China; 2 Fujian Provincial Key Laboratory of Neurodegenerative Disease and Aging Research, College of Medicine, Xiamen University, Xiamen, China; University of Tennessee, United States of America

## Abstract

Condensed tannins from *Ficus virens* leaves, fruit, and stem bark were isolated and their structures characterized by ^13^C nuclear magnetic resonance spectrometry, high performance liquid chromatography electrospray ionization mass spectrometry, and matrix-assisted laser desorption/ionization time-of-flight mass spectrometry. The results showed that the leaves, fruit, and stem bark condensed tannins were complex mixtures of homo- and heteropolymers of B-type procyanidins and prodelphinidins with degrees of polymerization up to hexamer, dodecamer, and pentadecamer, respectively. Antityrosinase activities of the condensed tannins were studied. The results indicated that the condensed tannins were potent tyrosinase inhibitors. The concentrations for the leaves, fruit, and stem bark condensed tannins leading to 50% enzyme activity were determined to be 131.67, 99.89, and 106.22 μg/ml on monophenolase activity, and 128.42, 43.07, and 74.27 μg/ml on diphenolase activity. The inhibition mechanism, type, and constants of the condensed tannins on the diphenolase activity were further investigated. The results indicated that the condensed tannins were reversible and mixed type inhibitors. Fluorescence quenching, copper interacting, and molecular docking techniques were utilized to unravel the molecular mechanisms of the inhibition. The results showed that the hydroxyl group on the B ring of the condensed tannins could chelate the dicopper irons of the enzyme. Moreover, the condensed tannins could reduce the enzyme product *o*-quinones into colourless compounds. These results would contribute to the development and design of antityrosinase agents.

## Introduction

**Figure 1:**
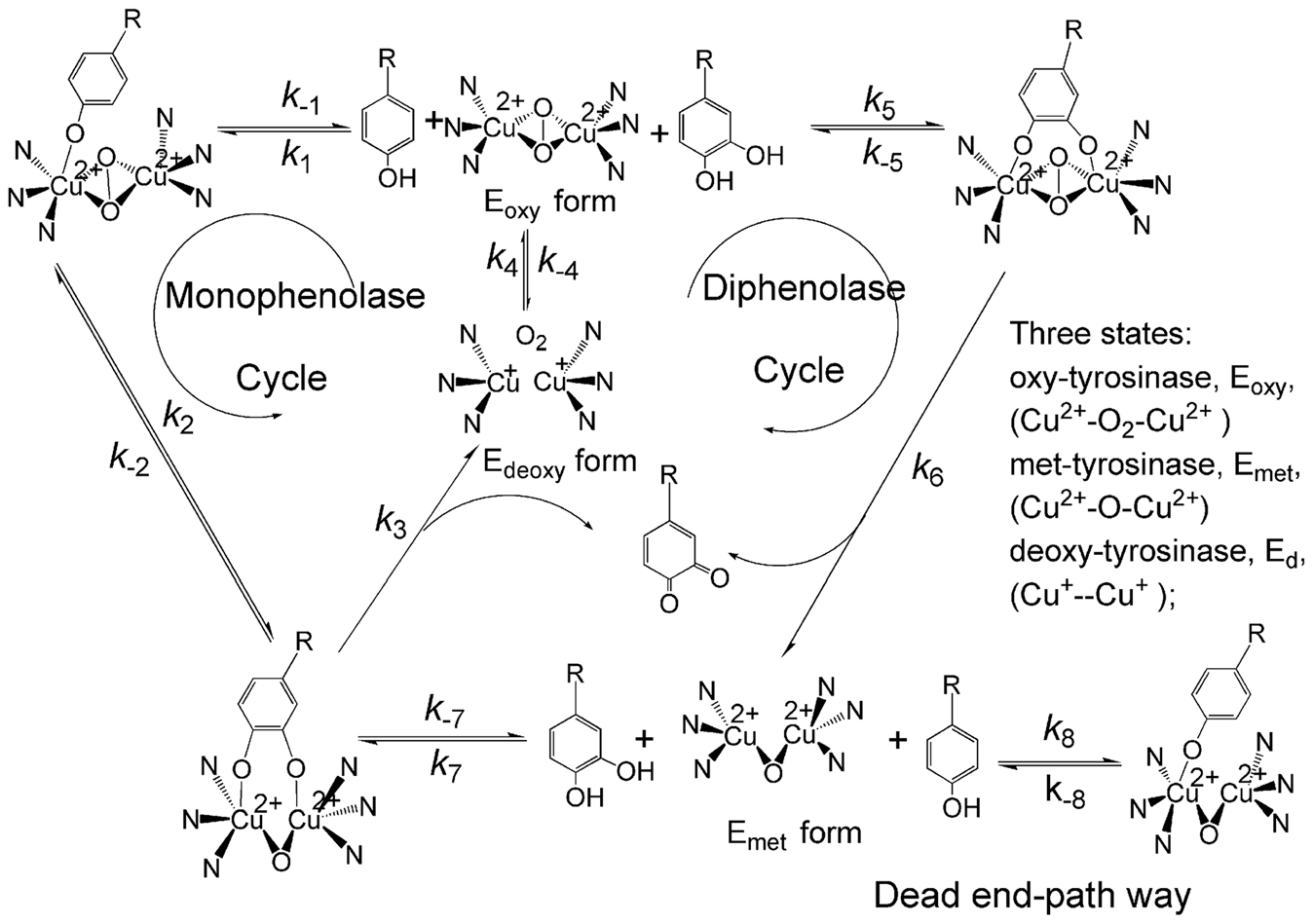
Catalytic cycles of the hydroxylation of monophenol and oxidation of *o*-diphenol to *o*-quinone by tyrosinase^2^.

Tyrosinase (EC 1.14.18.1) is a copper-containing, mixed-function oxidase widely distributed in animals, plants, and microorganisms [1]. Two copper ions of the enzyme at the active center are individually connected with three histidine residues and directly involved in different catalytic activities via oxy-, deoxy- and met-states (**Figure 1**) [2]. Consequently, the enzyme can catalyze both the hydroxylation of monophenols and the oxidation of *o*-diphenols into *o*-quinones [3]. The quinone product is a reactive precursor for the synthesis of melanin pigment. In humans, melanin-like pigments are synthesized by melanocytes predominantly within lysosome-like structure called melanosomes [4]. Normally, the presence and distribution of melanin are responsible for pigmentation formation in skin, hair and eyes of mammals. However, ultraviolet radiation, chronic inflammation, and the release of abnormal alpha-melanocyte stimulating hormone result in hyperpigmentation [5]. There is a wide array of targets against which to screen for depigmentation agents. One classic strategy for the treatment of abnormal pigmentation utilizes inhibitors of melanin synthesis enzyme. In human melanocytes, hydroxylation of L-tyrosine to L-dihydroxyphenylalanine (L-DOPA) by tyrosinase is an obligatory and rate-limiting step in melanogenesis. Thereafter, L-DOPA is oxidized by tyrosinase into dopaquinone, a common intermediate for both eu- and pheomelanogenic pathways [6], [7]. Over upregulated tyrosinase expression or activity is responsible for pigmentation disorders such as lentigo senilis, urticaria pigmentosa, age-related skin hyperpigmentation etc [7]. Thus, tyrosinase is an important target for the treatment of pigmentation disorders. Moreover, tyrosinase has also been reported to relate with Parkinson's disease and other neurodegenerative diseases [8], [9]. Therefore, tyrosinase inhibitors are quite significant in the area of cosmetic and medicinal industry, typically in the cosmetic industry where the development and screening of potent tyrosinase inhibitors are especially attractive.

**Figure 2:**
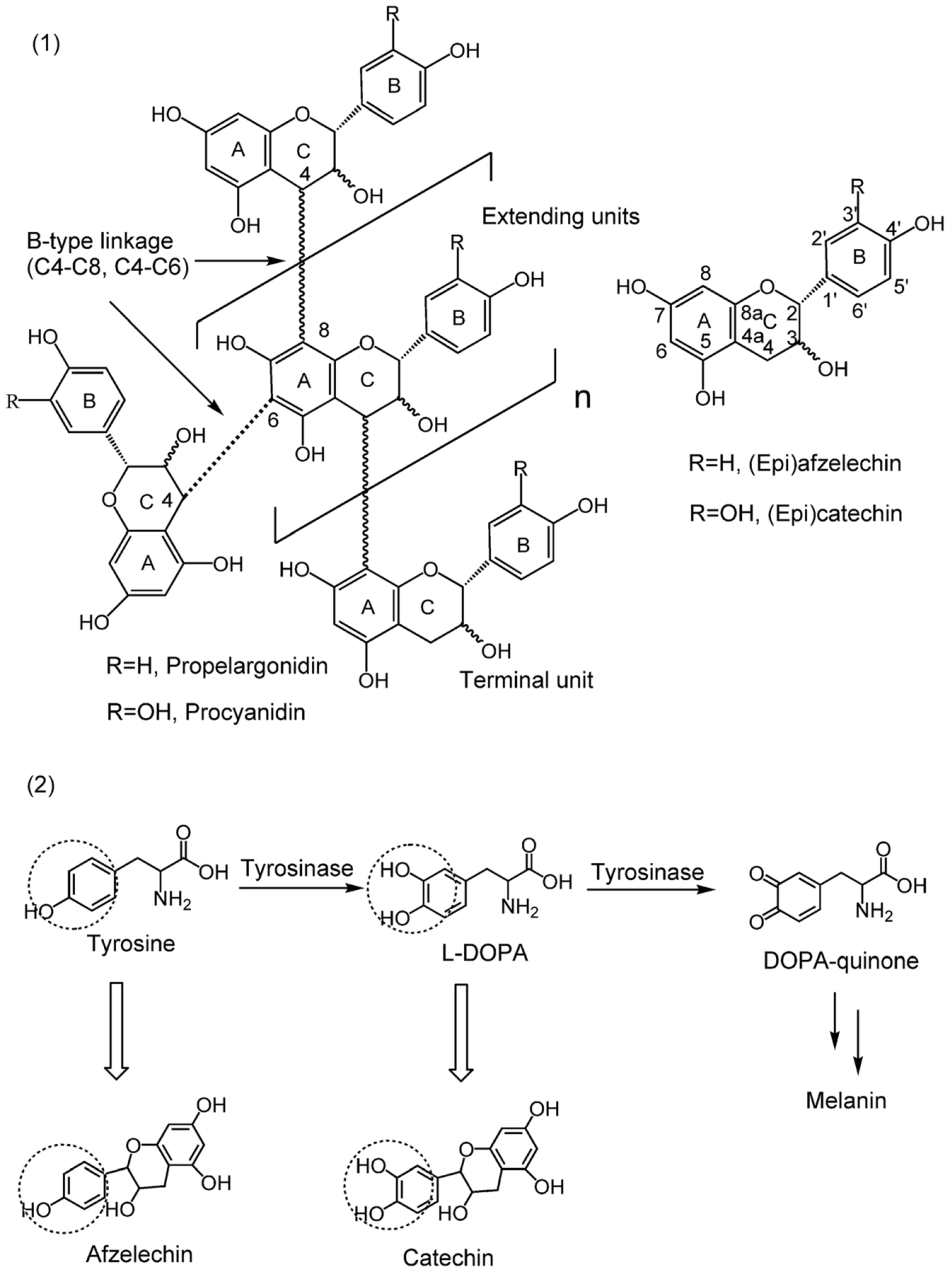
Structure of the condensed tannins, flavan-3-ol monomer units, and substrates of tyrosinase. (**1**) Chemical structure of the condensed tannins. The 4 to 6 linkage (dotted line) is an alternative interflavan bond. The terminal unit is at the bottom the multi-unit structure. (**2**) Structure similarities between the flavan-3-ol monomer units and the substrates of tyrosinase.

Condensed tannins have received considerable attention in the fields of nutrition, health and medicine, largely due to their physiological activities. Therefore, their biological activities such as antioxidant [10], anticancer [11], and antimicrobial activities are well documented [12]. Additionally, condensed tannins have also been reported to involve in the prevention of cardiovascular diseases [13]. Condensed tannins are composed of flavan-3-ol sub-units linked mainly through C4→C8 (or C4→C6) bonds (**Figure 2** **-1**) [14]. The structure diversity of condensed tannins derive from their different sub-units, interflavonoid bond positions, branching and the presence of non-flavonoid substituents such as gallic acid and sugars [15]. Furthermore, condensed tannins also vary markedly in molar mass distribution. For example, the condensed tannins in the plants may be composed of molecular species with a wide range of molar masses up to 20,000 [16]. Because of the complexity and diversity of their structures, structure characterization of condensed tannins still remains very challenging [17]. In this study, various techniques including ^13^C nuclear magnetic resonance (^13^C NMR), high performance liquid chromatography electrospray ionization mass spectrometry (HPLC-ESI-MS), and matrix-assisted laser desorption/ionization time-of-flight mass spectrometry (MALDI-TOF MS) were employed to characterize the structure of condensed tannins.

L-tyrosine (L-Tyr) and 3,4-dihydroxyphenylalanine (L-DOPA) are the substrates of tyrosinase and both of them have in common a phenolic group. As shown in **Figure 2-2**, catechin and afelzelcin, the monomer units of condensed tannins, are structurally similar with L-Tyr and L-DOPA, respectively. Moreover, Kim *et al.* (2006) [18] found that flavonoids, which are based on a common three-ring nucleus comprised of two benzene rings (A and B) linked through a heterocyclic pyran or pyrone ring in the middle, could inhibit tyrosinase activity by the interaction of the flavonoids with the copper ions in the catalytic domain of the enzyme. Furthermore, condensed tannins have also been reported to exhibit strong free radical scavenging activity [19]. On the bases of these information, we hypothesized that condensed tannins might exert antityrosinae activity by chelating the copper ions of the enzyme and scavenging the *o*-quinones generated by the enzyme.

*Ficus virens* is a medium sized tree belonging to the group of strangling figs which occurs by the stream side in subtropical China, tropical south, and south-east Asia. In the traditional medicine system, many parts of *F. virens* such as bark, latex, leaves and fruits are used in the treatment of blood diseases, apoplexy, vertigo, delirium, pain, rheumatism, diabetes and also as antioxidants [22]. Phytochemical investigations of *F. virens* leaves and stem bark revealed that phenolic compounds are their major components [20]–[23]. Moreover, a detailed survey of literature showed that tannins are widely distributed in various parts of this plant [24], [25].

Therefore, in this research the chemical structure, tyrosinase inhibitory activity and mechanism of inhibition of the condensed tannins from leaves, fruit, and stem bark of *F. virens* were studied. Their structures were established with the aid of ^13^C NMR spectroscopic, reverse phase HPLC-ESI-MS, and MALDI-TOF MS analyses. Kinetics analysis, fluorescence quenching, copper interaction, and molecular docking studies were performed to unravel the molecular mechanism of the inhibition on tyrosinase by the condensed tannins. To the best of our knowledge, this is the first report on the isolation and identification of the condensed tannins from the leaves, fruit, and stem bark *F. virens* and the elucidation of their antityrosinase activity and the mechanism of inhibition.

## Results and Discussion

### ^13^C NMR Analysis of the Condensed Tannins

**Figure 3:**
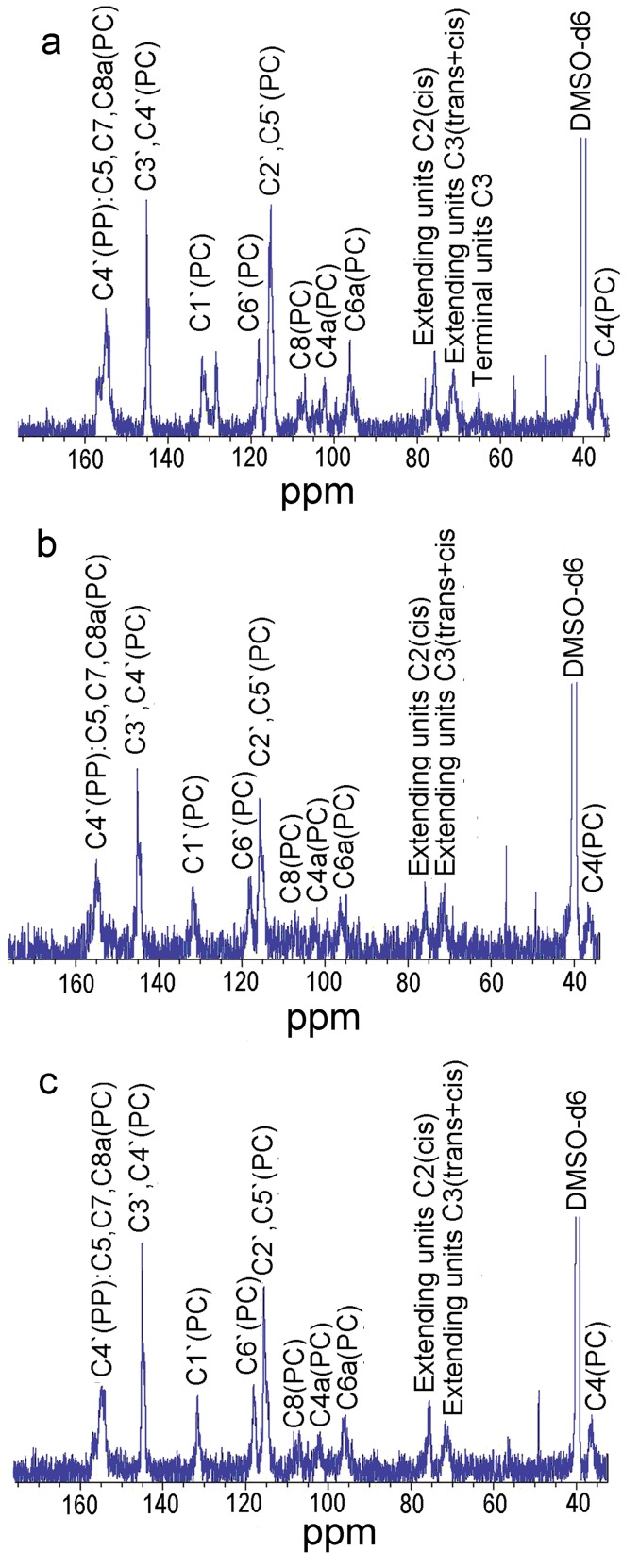
^13^C NMR (150 MHz) spectra of the condensed tannins in DMSO-*d*_6_. PC: procyanidin; PP: propelargonidin; **a**, **b**, and **c** represented the condensed tannins from leaves fruit, and stem bark of *F. virens*, respectively.

The ^13^C NMR spectra of the condensed tannin from the leaves (**a**), fruit (**b**), and stem bark (**c**) were analyzed and the results given in **Figure 3**. The signal assignment was with reference to our previous report [19]. The ^13^C NMR spectra showed the presence of procyanidin (PC) and propelargonidin (PP) for the leaves, fruit, and stem bark condensed tannins. The peaks between 70 and 90 ppm were used to determine the ratio of the 2,3-cis to 2,3-trans isomers through the distinct differences in their respective C2 chemical shifts. The C2 gave resonances of 76 ppm and 83 ppm for the cis and trans forms, respectively. In the present study, signal at 83 ppm was not detected indicating that the terminal units of the leaves, fruit, and stem bark condensed tannins were all in cis form (epicatechin). However, C3 of both cis and trans isomers occurred at 71.5 ppm. Besides, the resonance at 64 ppm was due to C3 of the terminal units. Therefore, the extender to terminal ratio of the leaves condensed tannins was estimated to be 4.18, whereas the ratios for the fruit and stem bark condensed tannins were not available because their terminal signals could not be detected.

### Thiolysis with Benzyl Mercaptan Followed by Reversed-phase HPLC-ESI-MS

**Figure 4:**
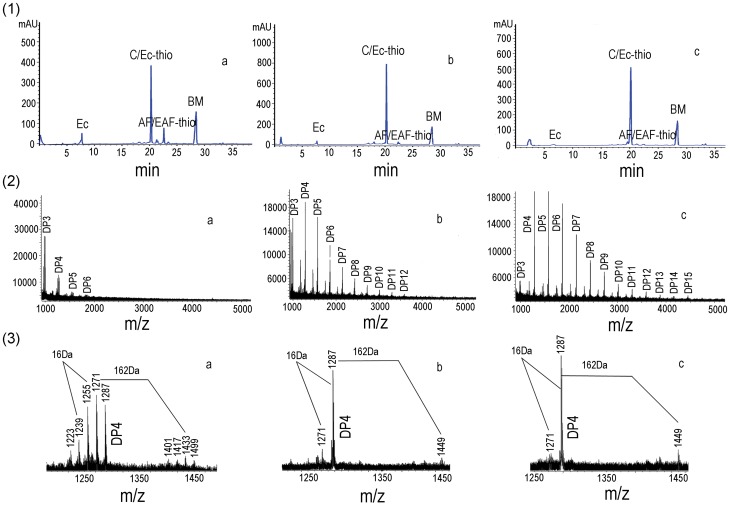
HPLC chromatograms and MALDI-TOF MS of the condensed tannins. (**1**) Reversed-phase HPLC chromatograms of the condensed tannins. Terminal units: epicatechin (EC). Extender units: afzelechin (AF-thio), epiafzelechin (EAF-thio), catechin (C-thio), epicatechin (EC-thio), BM, benzyl mercaptan. (**2**) MALDI-TOF positive reflectron mode mass spectra of the condensed tannins. DP, degree of polymerization. (**3**) Enlarged picture of the DP 4 of the condensed tannins. **a**, **b**, and **c** represented the condensed tannins from leaves fruit, and stem bark of *F. virens*, respectively.

Reversed-phase HPLC-ESI-MS analysis was utilized to obtain more detailed information on the chemical composition of the condensed tannins. The chromatograms of the thiolytic degraded condensed tannins from the leaves (**a**), fruit (**b**), and stem bark (**c**) were shown in **Figure 4** **-1**. The data showed that the extension units of the leaves, fruit, and stem bark condensed tannins all consisted of afzelechin/epi-afzelechin and catechin/epicatechin, with the catechin/epicatechin dominating. While the fruit and stem bark only contained a few afzelechin/epiafzelechin benzylthioethers compared with those of the leaves. Furthermore, the terminal units of the leaves, fruit, and stem bark condensed tannins were determined as epicatechin. Lastly, the mean degree of polymerization (DP) for the leaves, fruit, and stem bark condensed tannins were calculated to be 7.03, 15.95, and 40.41, respectively. Therefore, the results of the reversed-phase HPLC-ESI-MS were in consistent with those of the ^13^C NMR.

### MALDI-TOF MS Analysis

Although the ^13^C NMR spectra and reversed-phase HPLC-ESI-MS revealed complex structure characteristics of the condensed tannins, quantitative data regarding the high DP could not be reliably obtained. Further characterization was achieved by MALDI-TOF MS. In this research, MALDI-TOF MS of the condensed tannin were recorded as Cs^+^ adducts in the positive ion reflectron mode and showed repeating peaks (**Figure 4-2**). The polydisperse condensed tannin was reflected by the periodic occurrence of peak series representing different chain lengths. The masses of the highest peaks among the condensed tannin polymers with identical DP increased at a distance of 288 Da in the leaves, fruit, and stem bark condensed tannins corresponding to the addition of one catechin/epicatechin monomer unit. It could also be observed that the condensed tannins from *F. virens* had different polymer chain length with DP up to hexamer for the leaves, to dodecamer for the fruit, to pentadecamer for the stem bark (**Figure 4-2**). In addition to the predicted homopolyflavan-3-ol mass series mentioned above, each DP of the leaves, fruit, and stem bark condensed tannins had a subset of peak with mass 16 Da lower than the highest peak (**Figure 4-3**). These masses indicated the polymer chains contained one monomer unit with only one hydroxyl group (16 Da) on the aromatic ring B. Moreover, each DP of the leaves condensed tannins had several subsets of peaks with masses 16 Da lower than the highest peaks and the signals were relatively strong indicating that the leaves condensed tannins contained many afzelechin units (**Figure 4-3-a**). However, each DP in the spectra of the fruit and stem bark condensed tannins only had a subset of mass 16 Da lower than the highest peaks and the signals were relatively weak (**Figure 4-3-b** and **Figure 4-3-c**) indicating that the fruit and stem bark condensed tannins only contained a few afzelechin units. Thus, the leaves, fruit, and stem bark condensed tannins all contained PP and PC. These results indicated that the leaves, fruit, and stem bark condensed tannins were homo- and heteropolymers. Furthermore, each group of peaks was always followed by mass signals at a distance of 162 Da apart (corresponding to the addition of one glucose group at the heterocyclic C-ring) (**Figure 4-3**). In addition, no series of compounds that were 2 Da multiples lower than those described peaks for homo- and heteropolyflavan-3-ols were detected, so A-type interflavan ether linkage did not exist between adjacent flavan-3-ol subunits for the leaves, fruit, and stem bark condensed tannins. Therefore, all the condensed tannins were linked through B-type bonds.

### Effects of the Condensed Tannins on Monophenolase and Diphenolase Activities of Mushroom Tyrosinase

**Figure 5:**
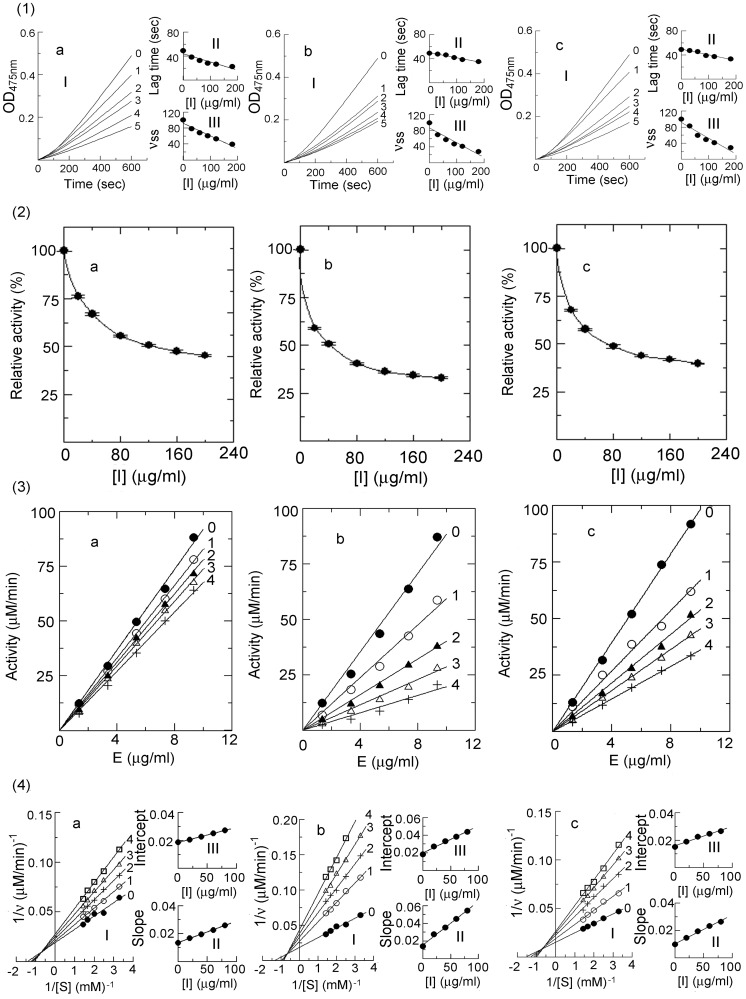
Determination of the inhibitory activity, inhibition mechanism, type, and constants. (**1**) Inhibitory activity of the condensed tannins on monophenolase activity of mushroom tyrosinase. (**I**) Progress curves for the oxidation of L-Tyr by the enzyme. (**II**) Effects on the oxidation rate of L-Tyr by the enzyme. (**III**) Effects on the lag time of monophenolase. The concentrations of the condensed tannins for curves **0**–**5** were 0, 30, 60, 90, 120, and 180 μg/ml, respectively. (**2**) Inhibitory activity of the condensed tannins on diphenolase activity of mushroom tyrosinase. (**3**) Inhibition mechanism of the condensed tannins on mushroom tyrosinase. The concentrations of the condensed tannins for curves **0**–**4** were 0, 20, 40, 60, and 80 μg/ml, respectively. (**4**) Determination of the inhibition type and constants of the condensed tannins on mushroom tyrosinase. (**I**) Lineweaver-Burk plots for diphenolase activity. (**II**) The plot of slope versus the concentration of the condensed tannins for determining the inhibition constants *K_I_.* (**III**) The plot of intercept versus the concentration of the condensed tannins for determining the inhibition constants *K_IS_*. Assay conditions: 3 ml reaction system containing 50 mM phosphate sodium buffer (pH 6.8) and 3.3% DMSO. **a**, **b**, and **c** represented the condensed tannins from leaves, fruit, and stem bark of *F. virens*, respectively.

The inhibition of the condensed tannins on the oxidation of L-Tyr by mushroom tyrosinase were assayed (**Figure 5-1**). The kinetics courses for the oxidation of the L-Tyr in the presence of the condensed tannins were shown in **Figure 5-1-I**. The results showed that the inhibition of the condensed tannins on the reaction rate of L-Tyr was dose-dependent, while the lag period of the enzyme reaction was shortened with increasing concentration of the condensed tannins (**Figure 5-1-II** and **Figure 5-1-III**). The concentrations for the leaves, fruit, and stem bark condensed tannins leading to 50% enzyme activity (*IC*_50_) were given in **Table 1**.

**Table 1:** Inhibition constants of condensed tannins from the leaves, fruit, and stem bark of *F. virens*.

Samples	*IC*_50_ (μg/ml)	*IC*_50_ (μg/ml)	Inhibition	Inhibition	Inhibition constants (μg/ml)	
	monophenolase	diphenolase	mechanism	type	*K_I_*	*K_IS_*
Leaves	131.67	128.42±0.45	Reversible	mixed	87.00	170.41
Fruit	99.89	43.07±0.62	Reversible	mixed	35.83	64.61
Stem bark	106.22	74.27±0.68	Reversible	mixed	48.20	111.99

The effects of the condensed tannins on the oxidation of L-DOPA catalyzed by mushroom tyrosinase were also investigated (**Figure 5** **-2**). With increasing concentrations of the condensed tannins, the activity of the enzyme was observably reduced. In this research, the *IC*_50_ values were estimated to be 128.42±0.45, 43.07±0.62, and 74.27±0.68 μg/ml for the leaves, fruit, and stem bark condensed tannins, respectively.

According to Khatib *et al.* (2007) [26], compounds showed *IC*_50_ values lower than 10 μM were considered as high potent inhibitors. Considering the high molecular mass of the condensed tannins, the fruit condensed tannins, which showed an *IC*_50_ of 99.89 μg/ml on the monophenolase activity of tyrosinase (rate-limiting reaction), may therefore be considered as an excellent tyrosinase inhibitor. As observed in **Table 1**, the condensed tannins were more effective in inhibiting the second step of oxidation (L-DOPA oxidation). The fact that the condensed tannins mainly consisted of catechin units which bears more resemblances to the structure of L-DOPA than L-Tyr could be the cause for their more potent inhibitory activity on the second stage of the enzyme reaction.

The lag time is known for the oxidation of monophenolic substrates such as oxidation of L-Tyr to L-DOPA. It had been reported that the lag time could be shortened or abolished by the presence of reducing agents known as cofactors, especially *o*-diphenols such as L-DOPA and catechin [27]. Moreover, many reducing agents, capable of reducing the E*_met_* form, shorten or abolish the lag time [28]. As observed in **Figure 5-1-III**, the condensed tannins from *F. virens* could diminish the lag time. This phenomenon might be partially explained by the chemical structure of the condensed tannins which were mainly composed of catechin units. Moreover, the condensed tannins obtained in our study also exhibited strong DPPH and ABTS free-radical scavenging activities (data no shown). Therefore, the reducing power of the condensed tannins might play a role in shortening the lag time.

### Inhibition Mechanism, Type, and Constants of the Condensed Tannins on Mushroom Tyrosinase

To ascertain how the condensed tannins behaved, the rate of dopachrome formation was monitored as a function of the enzyme and L-DOPA concentrations, respectively. From the plots of the remaining enzyme activity versus the enzyme concentration at different concentrations of the condensed tannins, a family of straight lines, which all passed through the origin, were obtained (**Figure 5-3**). The slopes of the lines descended with an increase of the concentrations of the condensed tannins, indicating that the presence of the condensed tannins did not bring down the amount of active enzyme but just lead to a decrease in the enzyme activity. Therefore, the inhibition of tyrosinase by the condensed tannins was reversible.

The plots of 1/*v* versus 1/[*S*] gave a family of straight lines with different slopes and intercepts, which crossed at the second quadrant, indicating that the condensed tannins from the leaves, fruit, and stem bark of *F. virens* were mixed type inhibitors (**Figure 5-4**). The results revealed that the condensed tannins bound with free enzyme as well as enzyme-substrate complexes. Furthermore, the inhibition constant (*K_I_*) were obtained from the plots of the slopes versus the concentrations of the condensed tannins, and the enzyme-substrate complex (*K_IS_*) were obtained from the vertical intercepts versus the concentrations of these compounds. The values of *K_I_* and *K_IS_* for the leaves, fruit, and stem bark condensed tannins were shown in **Table 1**. Since *K_IS_* were greater than *K_I_* for the oxidation of L-DOPA, the condensed tannins were able to bind more strongly to the free enzyme than to the enzyme-substrate complex. In summary, the results obtained from the kinetics analysis indicated that the condensed tannins were mixed type inhibitors, which meant that at the presence of the condensed tannins the affinity of the enzyme for the substrate was diminished.

### Fluorescence Quenching

**Figure 6:**
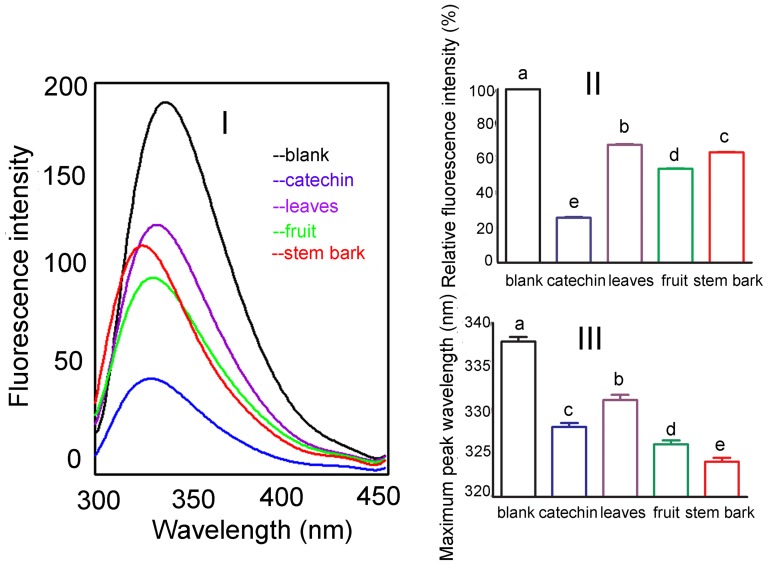
Changes in tyrosinase intrinsic fluorescence. The concentration of catechin and condensed tannins from the leaves, fruit, and stem bark was 85 μg/ml. (**I**) Intrinsic fluorescence changes. (**II**) Maximum fluorescence intensity changes. Values were expressed as mean±standard deviation (n = 3). (**III**) Maximum peak wavelength changes. Values were expressed as mean±standard deviation (n = 3). Different letters in the column denoted significantly different at P<0.05.

Fluorescence quenching study was performed to confirm the role played by the hydroxyl group of the condensed tannins in their inhibition on tyrosinase activities. The potent antityrosinase activity exerted by the condensed tannins might be ascribed to the B ring of the flavonol units, especially the -OH groups in positions 3′, 4′. This substitution pattern was reported to be responsible for the antityrosinase activity of flavonoids through their binding to the enzyme's active site [18]. Moreover, the study of Kim *et al*. (2006) [18] also revealed that the hydroxyl groups of the A and B rings on the 7, 3′,and 4′ positions primarily quenched the enzyme, and that the antityrosinase activity of the flavonoids was generated from their quenching of the enzyme. In this study, the interaction of the condensed tannins (catechin was used as a reference standard) with tyrosinase and the conformational alteration of the tyrosinase were evaluated by measuring the intrinsic fluorescence intensity of the enzyme before and after the addition of the condensed tannins. **Figure 6-I** showed that the presence of the condensed tannins caused dramatic changes in the fluorescence emission spectra. Besides, the fluorescence intensities of the emission peaks decreased in the order of catechin>fruit>stem bark>leaves (**Figure 6-II**). This phenomenon could be explained by the fact that the leaves condensed tannins contained more afzelechin units which only possessed one hydroxyl group at the 4′ position of the B ring. On the other hand, the condensed tannins from the fruit and stem bark contained more catechin units which possess two hydroxyl groups in the 3′, 4′ positions of the B ring. Furthermore, the decline in the fluorescence intensity was followed by *λ*em blue shift which indicated a more hydrophobic environment for the tryptophan residues of the enzyme. Interestingly, the range of *λ*em blue shift ranged in the order of stem bark >fruit> catechin >leaves (**Figure 6-III**). This might be ascribed to the fact that the stem bark condensed tannins had higher mean DP than those of the fruit and leaves condensed tannins. However, catechin had greater *λ*em blue shift than that of the leaves condensed tannins which might be because that the latter constituted a high percentage of afzelchin units. Taking the extent of the changes in the fluorescence intensities and *λ*em blue shift into consideration, the conclusion that both the number of hydroxyl groups in the B ring and the mean DP of the condensed tannins played a synergestic role in changing the conformation of the enzyme could be reached.

### The Chelating Ability of the Condensed Tannins

**Figure 7:**
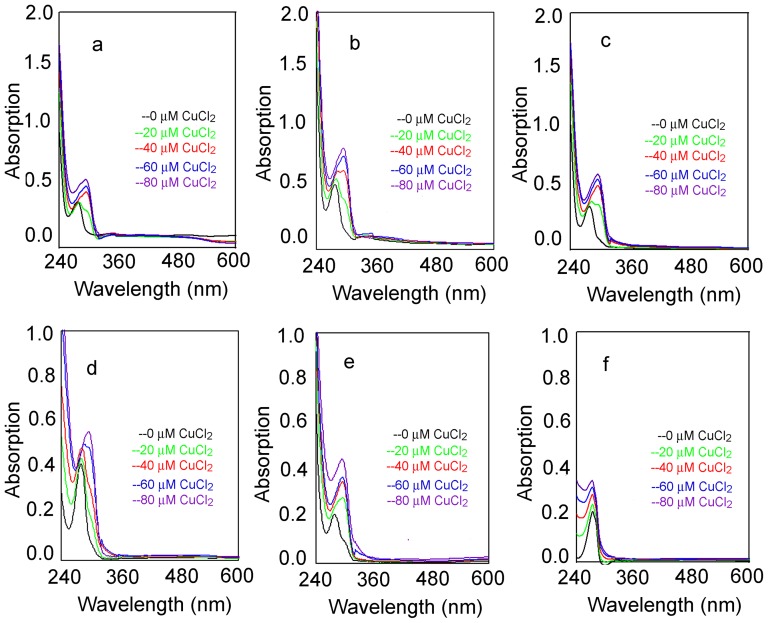
Absorption spectra for test samples and the Cu-test samples complex at pH 7.4. **a**, **b**, and **c**, represented the condensed tannins from the leaves, fruit, and stem bark of *F. virens*; **d**, **e**, and **f** represented catechin, L-DOPA, and L-Try.

From the fluorescence quenching study the hydroxyl groups of the condensed tannins were found to quench the intrinsic fluorescence of the tyrosinase. Moreover, condensed tannins have also been reported to chelate metal ions [29]. Therefore, it was examined whether the condensed tannins could chelate the copper ions which are a metal constituent in the catalytic domain of the enzyme. In this research, the direct interaction of the condensed tannins with the Cu^2+^ ions at pH 7.4 was assessed by UV-visible spectroscopy. The interaction between the condensed tannins and the copper ions showed various significant red shift peaks (**Figure 7**). For example, the peaks at 278 nm were red-shifted to 295 nm as the condensed tannins bound to the copper ions indicating the formation of chelate between the condensed tannins and the copper ions (**Figure 7-a**, **Figure 7-b**, and **Figure 7-c**). At the same time, L-DOPA and catechin showed similar peak shifts with those of the condensed tannins (**Figure 7-d** and **Figure 7-e**). However, L-Tyr with mono-hydroxyl structure in the aromatic ring only exhibited increasing absorbance at 273 nm without any wavelength shifts (**Figure 7-f**). Therefore, it was concluded that these inherent characteristics of the condensed tannins to form chelates with the copper ions inhibited the tyrosinase by forming an interaction between the condensed tannins and copper ions and the enzyme catalytic site.

### Molecular Docking Analysis

**Figure 8:**
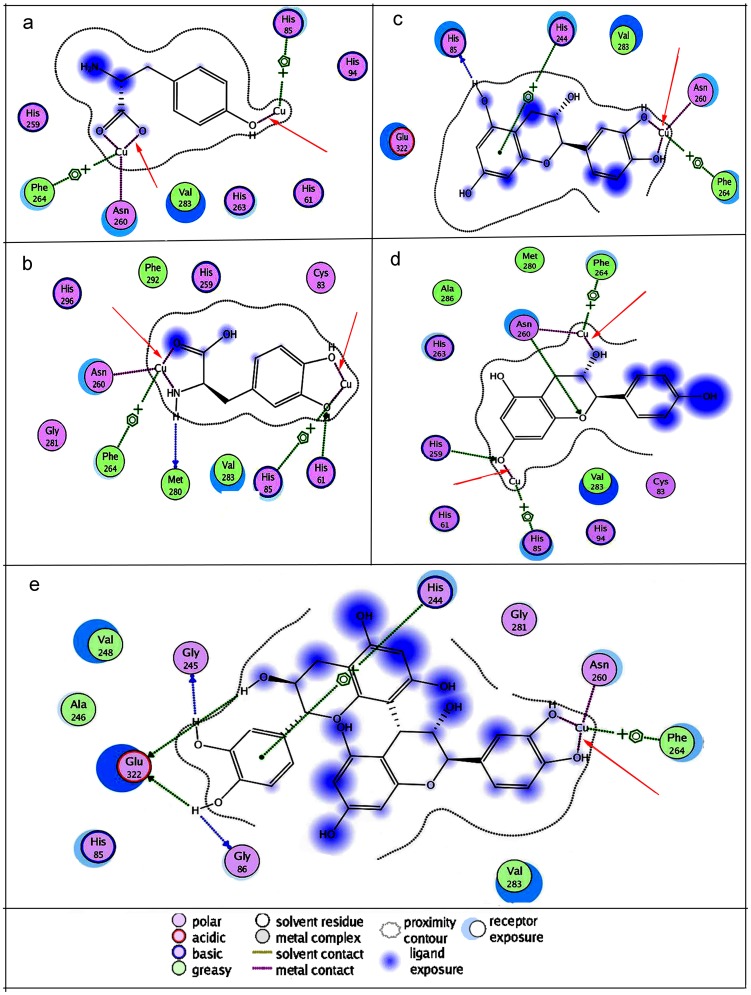
Binding mode of the lowest energy docked conformation found for the ligand with tyrosinase residues. The receptor exposure differences were shown by the size and intensity of the turquoise discs surrounding the residues. The red arrows indicated the interaction of the ligand with the copper iron. **a**, **b**, **c**, **d**, and **e** represented L-Tyr, L-DOPA, catechin, afzelechin, and PCs dimmers, respectively.

Docking simulations were further performed to understand the mechanism underlying the potent antityrosinase activities of the condensed tannins. The docking modes of L-Tyr, L-DOPA, monomers of the condensed tannins (catechin and afzelelcin), and dimmers of PC were examined in the enzyme catalytic site. The docked conformations revealed that L-Tyr, L-DOPA, catechin, afzelelcin, and PC dimmers could form metal interactions with the dicopper irons of the enzyme (**Figure 8**). Interestingly, the dihydroxyl group of the L-DOPA, catechin and PC dimmers could also chelate the copper ions in the enzyme active site (**Figure 8-b**, **Figure 8-c**, and **Figure 8-e**), whereas L-Tyr and afzelelcin could not chelate the copper ions (**Figure 8-a** and **Figure 8-d**). The results corresponded with those of copper interaction study. By combining the results of copper interaction and molecular docking studies, the conclusion that the condensed tannins had copper chelating ability, which might enhance their inhibitory potency on the enzyme could be comfortably reached.

### Interaction of the Condensed Tannins with *o*-Quinones

**Figure 9:**
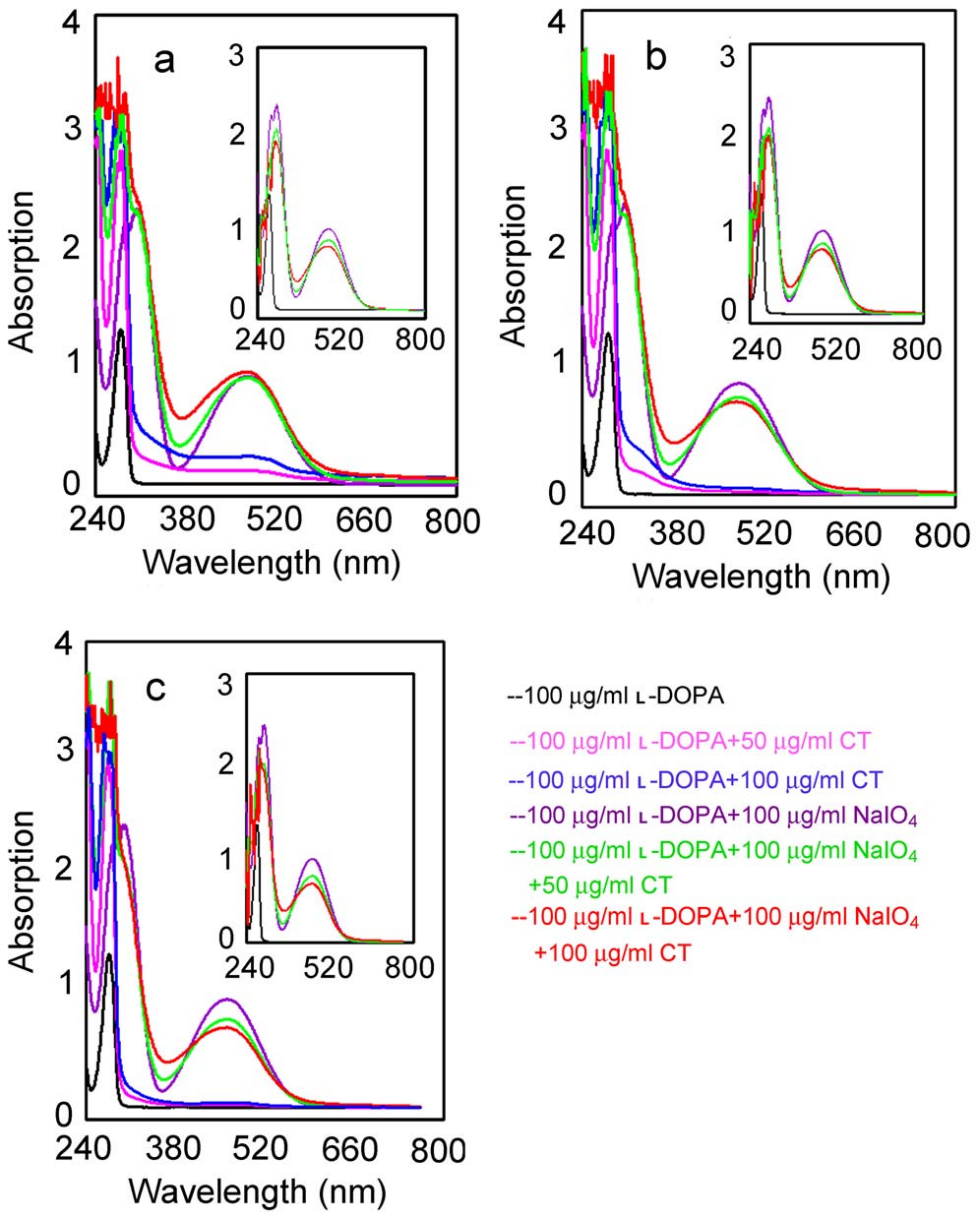
UV-Vis spectra for the oxidation of L-DOPA. **Black**, 100 μg/ml L-DOPA; **Pink**, 100 μg/ml L-DOPA+50 μg/ml CT; **Blue**, 100 μg/ml L-DOPA +100 μg/ml CT; **Purple**, 100 μg/ml L-DOPA+100 μg/ml NaIO_4_; **Green**, 100 μg/ml L-DOPA+100 μg/ml NaIO_4_+50 μg/ml CT; **Red**, 100 μg/ml L-DOPA+ 100 μg/ml NaIO_4_+100 μg/ml CT. The green and red spectra of the insert is corresponding to the green and red spectra of the main figure after subtraction of the corresponding condensed tannins absorption from the spectra, while the purple spectra of the insert is the same as the purple in the main figure. **a**, leaves condensed tannins; **b**, fruit condensed tannins; **c**, stem bark condensed tannins; CT, condensed tannins. The assay was performed in 3 ml of 50 mM sodium phosphate buffer (pH 6.8) at 30°C.

To elucidate whether the condensed tannins could directly form oxidoreduction reactions toward *o*-quinones with regeneration of the corresponding *o*-diphenol, the *o*-diphenol (L-DOPA) was oxidized by sodium-periodate (NaIO_4_) in the absence and presence of the condensed tannins. L-DOPA alone did not show any characteristic peaks (**Figure 9**, **black**), while the oxidation of L-DOPA by NaIO_4_ gave rises to the corresponding *o*-quinones with a characteristic peak at 475 nm (**Figure 9**, **purple**). However, when the condensed tannins were added, a decrease of absorbance at the peak occurred (inserts of **Figure 9**, **green** and **red**). Therefore, the reducing power of the condensed tannins might decrease the formation of *o*-quinones by oxidoreduction reaction toward the *o*-quinones. It is known that *o*-dopaquinone undergoes a fast intramolecular cyclization to yield leukodopachrome which is oxidized by another molecule of *o*-dopaquinone to render dopachrome [30]. This aminechrome is relatively stable in the medium although it may eventually be converted into melanins. The result of this study could be explained by the hypothesis that the condensed tannins could not directly interact with dopachrome, however, it could compete by means of nucleophilic attack with the internal cyclization, thus preventing dopachrome formation. Therefore, when L-DOPA was oxidized by the NaIO_4_, the condensed tannins were already present in the medium to interact with *o*-dopaquinone before the cyclization took place.

In conclusion, the structures of the condensed tannins from the leaves, fruit, and stem bark of *F. virens* were successfully characterized. Furthermore, the condensed tannins exhibited great efficiency in inhibiting both mono- and diphenolase activities of the mushroom tyrosinase, and were found to be mixed type inhibitors of the enzyme. Moreover, the inhibition was carried out mainly through the interaction of the hydroxyl groups in the aromatic ring B of the condensed tannins with the active center of the enzyme. Additionally, the condensed tannins could directly scavenge the *o*-quinones through oxidoreduction reaction. The elucidation of the molecular mechanisms of the antityrosinase activities of the condensed tannins is significant in both designing and screening of potent novel tyrosinase inhibitiors. However, in order to develop therapeutic agent for treating abnormal skin pigmentation, the effects of the condensed tannins on human tyrosinase activities needs further research.

## Methods

### Plant Materials

The leaves, fruit, and stem bark of *F. virens* were collected at the campus of Xiamen University (Xiamen, China) and immediately freeze-dried and ground using a cutting mill (model BL301D5; Saikang, China) to pass through 100 mesh sieves to obtain fine powder. The resulting powder was stored at −20°C in a freezer prior to further processes.

### Chemistry

Mushroom tyrosinase (EC 1.14.18.1) was the product of Sigma Chemical Co. (St. Louis, MO, USA) with specific activity of 6680 U/mg. L-Tyr, L-DOPA, dimethylsulfoxide (DMSO), catechin/epicatechin, Sephadex LH-20, benzylmercaptan, Amberlite IRP-64 cation-exchange resin, and cesium chloride were purchased from Sigma Aldrich (USA). All analytical grade solvents, including acetone, n-hexane, petroleum ether, ethyl acetate and methanol, and HPLC-grade dichloromethane, acetonitrile (CH_3_CN), methanol, and trifluoroacetic acid (TFA) were obtained from Sinopharm (Sinopharm, Shanghai, China). Water used in this study was purified on a Millipore Milli-Q apparatus (TGI Pure 110 Water Systems, USA).

### Extraction and Purification of the Condensed Tannins

Freeze-dried leaves, fruit, and stem bark powders (20 g of each) were extracted three times with 70% (v/v) acetone (3×150 ml) at room temperature. Each extract was filtered and pooled, and the acetone was eliminated by evaporation under vacuum (38°C). The remaining aqueous was defatted with petroleum ether (3×150 ml) to remove chlorophyll and lipophilic compounds, followed by extraction with ethyl acetate (3×150 ml) to remove low molecular phenolics. The remaining aqueous was freeze-dried to obtain crude tannin. Next, the crude tannin was chromatographed on a LH-20 column which was first eluted with methanol-water (50∶50, v/v) and then with acetone-water (7∶3, v/v). The acetone-water fraction was collected and the acetone removed by rotary evaporation, and then the aqueous fraction freeze-dried to obtain the condensed tannins.

### ^13^C NMR Analysis

For recording ^13^C NMR spectra, the condensed tannins were dissolved in DMSO-*d*_6_. The ^13^C NMR spectra of the condensed tannins were recorded on a Varian Mercury-600 spectrometer (Palo Alto, CA, USA) at 150 MHz as described in reference [31].

### Thiolysis of the Condensed Tannins with Benzyl Mercaptan

The condensed tannins (500 μg, dissolved in methanol) were mixed with 3.3% hydrochloric acid methanol solution and 5% benzyl mercaptan methanol solution. The mixed solution was heated at 40°C for 30 min, and then cooled to room temperature. Then, the solution was filtered through a membrane filter (aperture size: 0.22 μm) to obtain the thiolysis medium.

### Reversed-phase HPLC-ESI-MS Analysis

The high performance liquid chromatograph was an Agilent 1100 system (Agilent, Santa Clara, CA, USA) equipped with a diode array detector and a quaternary pump. The thiolysis medium was further analyzed using LC/MS (QTRAP 3200, USA) with a Hypersil ODS column (4.6×250 mm) (Elite, Dalian, Liaoning Province, China). The experiment protocol was described in our previous study [19].

### MALDI-TOF MS Analysis

The MALDI-TOF MS spectra were recorded on Bruker Reflex III (Germany). The experiment was carried out with reference to the method described by Xiang *et al*. (2006) [32]. 2,5-Dihydroxybenzoic acid (DHB, 10 mg/ml 30% acetone solution) was used as matrix. Amberlite IRP-64 cation-exchange resin (Sigma-Aldrich, USA), equilibrated in deionized water, was used to deionize the analyte, matrix, and cesium chloride solution. The sample solutions (10 mg/ml 30% acetone solution) were mixed with the cesium chloride (1.52 mg/ml) solution at a volumetric ratio of 1∶1 to promote the formation of a single type of ion adduct ([M+Cs]^+^). Next, the mixture was mixed with the matrix solution (1∶3, v/v). Then, the mixture (1 μl) was spotted to the steel target.

### Enzyme Assay

In this investigation, L-Tyr and L-DOPA were used as substrates for monophenolase and diphenolase activities assays, respectively. The final concentrations of mushroom tyrosinase were 16.67 and 3.33 μg/ml for the determination of monophenolase and diphenolase activities. The procedures for tyrosinase activity assay were performed as described in our previous reported [33].

The inhibitory effects of the condensed tannins were expressed as the concentrations that inhibited 50% of the enzyme activity (*IC*_50_). The inhibition type was determined by the Line-weaver Burk plot, and the inhibition constant was determined by the secondary plots of the apparent *K*_m_/*V*_m_ or 1/*V*_m_ versus the concentration of the condensed tannins.

### Fluorescence Quenching

In this research, the fluorescence intensities were recorded using a Cary Eclipse fluorescence spectrophotometer. The concentration of catechin and the leaves, fruit, and stem bark condensed tannins was 85 μg/ml. The experiment was performed according to the protocol described in our previous study [34].

### Copper Interaction

To investigate the interaction between the copper ions and the condensed tannins, 30 μg/ml samples were prepared in cuvettes containing phosphate buffer (10 mM, pH 7.4). Scans were taken 10 seconds after the addition of CuCl_2_ to the samples using a DU800 spectrophotometer (Beckman Coulter Inc., CA). The absorption spectra were recorded between 240 and 600 nm.

### In Silico Docking of Tyrosinase with the Ligands

In this research, molecular operation environment 2010 software (MOE) was used for protein-ligand docking. As with other recent studies [35], [36], the structure of the oxy tyrosinase from *Streptomyces castaneoglobisporus* was used as the initial model for docking simulations after removal of the caddie protein, the exogenous ions, and water molecules (pdb entry 1wx2) [37]. The 3D structures of the ligands were prepared with Chembiodraw Utra 12.0. Before docking, the structure models of the protein and ligands were energy minimized using the energy minimization module of the MOE. At the same time, hydrogens were added to the models of the protein and ligands with the protonate 3D module.

For molecular docking, the refinement module was set to forcefield and retain of both first and second scoring were set to 10. The MM/GBVI binding free energy scoring was used to rank the docking poses. A more negative value reflects a stronger interaction. Other parameters used were the default settings of the software. The docked conformation which had the highest score was selected to analyze the mode of binding.

### Non-enzymatic Generation of the Quinones

The experiment was carried out with reference to the method of Espin and Wichers (2001) [38] L-DOPA (100 μg/ml) was oxidized by NaIO_4_ (100 μg/ml). The spectra obtained in the absence and presence of the condensed tannins (50 and 100 μg/ml) were recorded with the DU800 spectrophotometer.

### Statistical Analysis

The SPSS statistical package version 19.0 was used for calculations (SPSS, version 19.0, Inc.,Chicago, IL, USA). The data collected had a mean standard error (n = 3). Comparisons between samples were analyzed with one-way ANOVA, followed by Tukey's *post hoc* test. Differences were considered significant at *P*<0.05 and identified by different letters in figures.
